# IRAK2, an IL1R/TLR Immune Mediator, Enhances Radiosensitivity *via* Modulating Caspase 8/3-Mediated Apoptosis in Oral Squamous Cell Carcinoma

**DOI:** 10.3389/fonc.2021.647175

**Published:** 2021-06-23

**Authors:** Chih-Chia Yu, Michael W.Y. Chan, Hon-Yi Lin, Wen-Yen Chiou, Ru-Inn Lin, Chien-An Chen, Moon-Sing Lee, Chen-Lin Chi, Liang-Cheng Chen, Li-Wen Huang, Chia-Hui Chew, Feng-Chun Hsu, Hsuan-Ju Yang, Shih-Kai Hung

**Affiliations:** ^1^ Department of Medical Research, Dalin Tzu Chi Hospital, Buddhist Tzu Chi Medical Foundation, Chia-Yi, Taiwan; ^2^ Department of Radiation Oncology, Dalin Tzu Chi Hospital, Buddhist Tzu Chi Medical Foundation, Chia-Yi, Taiwan; ^3^ Research Center for Environmental Medicine, Kaohsiung Medical University, Kaohsiung, Taiwan; ^4^ Department of Biomedical Sciences, National Chung Cheng University, Chia-Yi, Taiwan; ^5^ Epigenomics and Human Disease Research Center, National Chung Cheng University, Chia-Yi, Taiwan; ^6^ Center for Innovative Research on Aging Society (CIRAS), National Chung Cheng University, Chia-Yi, Taiwan; ^7^ School of Medicine, Tzu Chi University, Hualian, Taiwan; ^8^ Department of Radiation Oncology, Zhongxing Branch, Taipei City Hospital, Taipei, Taiwan; ^9^ Department of Pathology, Chiayi Chang Gung Memorial Hospital, Chia-Yi, Taiwan

**Keywords:** IRAK2, radioresistant, apoptosis, radiosensitization, oral squamous cell carcinoma

## Abstract

Predicting and overcoming radioresistance are crucial in radiation oncology, including in managing oral squamous cell carcinoma (OSCC). First, we used RNA-sequence to compare expression profiles of parent OML1 and radioresistant OML1-R OSCC cells in order to select candidate genes responsible for radiation sensitivity. We identified IRAK2, a key immune mediator of the IL-1R/TLR signaling, as a potential target in investigating radiosensitivity. In four OSCC cell lines, we observed that intrinsically low IRAK2 expression demonstrated a radioresistant phenotype (i.e., OML1-R and SCC4), and vice versa (i.e., OML1 and SCC25). Next, we overexpressed IRAK2 in low IRAK2-expression OSCC cells and knocked it down in high IRAK2-expression cells to examine changes of irradiation response. After ionizing radiation (IR) exposure, IRAK2 overexpression enhanced the radiosensitivity of radioresistant cells and synergistically suppressed OSCC cell growth both *in vitro* and *in vivo*, and vice versa. We found that IRAK2 overexpression restored and enhanced radiosensitivity by enhancing IR-induced cell killing *via* caspase-8/3-dependent apoptosis. OSCC patients with high IRAK2 expression had better post-irradiation local control than those with low expression (i.e., 87.4% *vs.* 60.0% at five years, P = 0.055), showing that IRAK2 expression was associated with post-radiation recurrence. Multivariate analysis confirmed high IRAK2 expression as an independent predictor for local control (HR, 0.11; 95% CI, 0.016 – 0.760; P = 0.025). In conclusion, IRAK2 enhances radiosensitivity, *via* modulating caspase 8/3-medicated apoptosis, potentially playing double roles as a predictive biomarker and a novel therapeutic target in OSCC.

## Introduction

Radiotherapy (RT) is an essential treatment modality for managing patients with oral squamous cell carcinoma (OSCC) (1). However, cancer radioresistance restricts the clinical efficacy of RT. Although several genes and molecular pathways have been reported (2, 3), the molecular events leading to a radioresistant phenotype of OSCC remain mostly unknown. Therefore, exploring a novel targeted molecular marker that sensitizes tumors to ionizing radiation (IR) is crucial to overcome radioresistance and then decrease post-RT cancer recurrence.

RNA Sequencing (RNA-Seq) technology, widely used in studying whole-genome expression profiles, can help identify possible therapeutic targets (4). To search for genes potentially responsible for OSCC resistance that could predict radiosensitivity, we recently established a stable, radioresistant oral cancer cell subline (i.e., OML1-R) from its parent line (i.e., OML1) *via* step-by-step fractionated irradiations (5). Subsequently, we performed next-generation sequencing (NGS) and bioinformatics techniques to analyze post-IR gene expression between the two cell lines. Finally, we identified that IRAK2 was up-regulated in post-irradiated parental OML1 cells, but not in radioresistant OML1-R cells, implicating that the IRAK2 gene might play a role in the process of radiosensitivity in OSCC.

IRAK2 (Interleukin-1 receptor associated kinase 2) is a component of the interleukin-1 receptor (IL-1R)/Toll-like receptor (TLR) signaling cascade (6). Known to act as an adaptor in the TLR-MyD88-TRAF6 complex, IRAK2 could enable the downstream activation of NF-κB and thereby regulating inflammation (7). Notably, IRAK2 also participates in the regulation of cellular apoptosis *via* inducing the FADD-dependent caspase-8 apoptotic pathway to trigger the Yersinia-induced macrophage cell death (8). Besides, IRAK2 has been recognized as a contributor to ER stress-induced cell death *via* IRE1/CHOP signaling (9). Recently, one family member of IRAK2, i.e., IRAK1, has been reported to play a role in the processes of TLR signaling (10) and intrinsic radioresistance, suggesting a potential chemoradiotherapy target (11). However, the function and biologic effects of IRAK2 in association with intrinsic/acquired radioresistance in the context of solid cancers, including OSCC, remain mostly unknown.

Hence, in the present study, we present new insight into the significance of IRAK2 in radiation response, therefore, tested the role of IRAK2 in OSCC, focusing on exploring its potential function and molecular mechanism in mediating radiosensitization. Our data indicated that IRAK2 is an attractive target, in both predictive and therapeutic aspects, for radioresistant OSCC because the overexpression of IRAK2 may contribute to enhanced/restore IR-induced tumor cell killing.

## Materials and Methods

### Chemicals and Reagents

Antibodies against IRAK2, cleaved caspase-8, cleaved caspase-3, NF-κB-p65, and C/EBP homologous protein (CHOP) were purchased from the Cell Signaling Technology (Beverly, MA). Processes of storage, manipulation, and analysis obeyed the manufacturer’s instructions.

### Cell Lines and Cell Culture

SCC4 and SCC25 were bought from American Type Culture Collection (ATCC; Manassas, VA) and cultured in DMEM/F12 containing 10% fetal bovine serum (FBS), 1% penicillin-streptomycin and 2 mM L-glutamine. Parental (OML1) and acquired-radioresistant (OML1-R) cell lines were established and maintained in RPMI1640 containing 10% FBS, 1% penicillin-streptomycin, and 2 mM L-glutamine, as previously reported (5, 12). Briefly, we constructed OML-1R from parental OML-1 cells by using fractionated irradiations. A fraction size of 5 Gy was delivered per 5-7 days till every 80% confluent of irradiated cells. By ten fractions, a total of 50 Gy were delivered on parental OML-1 cells to construct OML-1R cells. Then, we applied a 10-Gy single shot to validate the level of acquired radioresistance of OML-1R cells before further experiments [10]. Briefly, a total of 1 x 10^3^ cells were seeded on a 6-cm plate before irradiation. The next day, cells were irradiated with 10 Gy and then cultured for another 2-3 days.

### Patient Samples and Radiotherapy Details

From Jan. 2007 to Dec. 2014, we retrospectively identified 41 patients with pathological stage I-II OSCC (i.e., pT1-2N0M0 status) (12, 13). The reason to choose this population was that OSCC patients with microscopic residual disease (R1) or close surgical margins of ≤5 mm (R0) have an increased risk for local failure even if the resected tumor was staged as pT1-2N0M0 and therefore were subjected to postoperative RT according to guidelines (1). All patients received radical surgery and postoperative RT. Indications of RT for these patients were positive or close surgical margin (i.e., ≤5 mm), as mentioned previously (13). All OSCC patient samples (i.e., formalin-fixed paraffin-embedded pathological blocks) were retrospectively re-confirmed, and tumor-burden-enriched regions (i.e., >70% tumor-content area) were re-sliced, re-stained, and retrieved for bench experiments, as previously reported (12). Postoperative RT was delivered using the intensity-modulated radiotherapy (IMRT) technique (14). Irradiation volumes were designed according to the principle of radiotherapy (15), in terms of high-, moderate-, and low-risk planning target volume (PTV). Notably, total doses of RT to the high-risk PTV (i.e., the oral surgical bed and high-risk lymph-drainage basins) were ranged from 60 Gy to 66 Gy by using a conventional fraction size of 1.8-2.0 Gy (6-MV photons).

### Research Database of Clinical Outcomes

For coding post-irradiation clinical outcomes, we used the Dalin Prospective-coding Cancer Registration Database. This database was a regular national-audit cancer database for oncological research. Regular audits were conducted by the multimodality committee of the Health Promotion Administration, Ministry of Health and Welfare, Taiwan (16). At the latest audit in 2018, the overall data-consisting rate was 99.5%. For each involved patient, the following clinicopathological factors were retrospectively retrieved from the database: age, RT dose, pathologic stage, clinical stage, surgical margin, and postoperative adjuvant chemotherapy (17–19). All data were independently validated by a radiation physician and analyzed by a biostatistician according to methods described in the statistical section, as previously reported (12).

### Illumina MiSeq System

We used TRIZOL to isolate total RNAs according to the manufacturer’s instructions (Invitrogen, Carlsbad, CA). Next, we used the Illumina MiSeq (Illumina, San Diego, CA) to conduct RNA-Seq. The mapped reads (i.e., Reads Per Kilobase Million [RPKM]) were applied to indicate gene expression levels; this value was used to calculate the average expression level for each gene between paired OML1 and OML1-R cell lines treated with or without IR. Gene expression profiles of both cells were obtained from the Gene Expression Omnibus (GEO) database (https://www.ncbi.nlm.nih.gov/geo/query/acc.cgi?acc=GSE165585). First, we selected genes that exhibited a statistically significant difference of higher than 1.5-fold between OML1 and OML1-R cells after IR. Next, we filtered out lowly expressed transcripts by using an absolute value of RPKM < 2. Then, we identified eight genes of IRAK2, KLK6, NSMF, SCO1, TRIP13, LMBR1, SCARB1, and FANCD2. Finally, we investigated IRAK2 because IRAK2 showed the maximum fold change of gene expression.

### Colony Formation Assay

OSCC cell lines were treated with indicated irradiation doses of 0, 4, or 10 Gray (Gy) by using 6-MV photons (Varian linear accelerator, US), as previously reported (12). Notably, to provide effective dose delivery, a 0.5-cm bolus was placed over both the upper and downsides of the culture dishes, just like a sandwich design (5). Briefly, cells were cultured in a specific medium for more than 80% fluency. Next, we trypsinized and plated the cells to produce a single-cell suspension in another culture dish. Then, irradiation was delivered per protocol in the irradiating arm; the control arm had no irradiation. At seven days after irradiation, colonies (defined as groups of >50 cells) were fixed and stained with 0.05% crystal violet for further visual quantification. For quantifying cell number, irradiated cells were stained with 0.4% crystal violet (Sigma) and counted at OD580 by using a spectrophotometer (GeneQuant 1300, GE Healthcare, UK) (20).

### Western Blotting

We lysed cells with 100μl of PRO-PREP Protein Extraction Solution according to the manufacturer’s protocol. Then, protein samples (50 µg/well) were separated by 12% SDS-PAGE electrophoresis and transferred to PVDF membranes (at 260 mA for about 90 minutes), as reported previously (21). Briefly, membranes were blocked with 5% non-fat dried milk in 1X TBS-T buffer (for 1 hour at room temperature) and probed with primary antibodies (diluted with 5% non-fat milk in 1X TBST), followed by HRP-labeled secondary antibodies (also diluted with 5% non-fat milk in 1X TBST). Finally, bands were visualized by using electrochemiluminescence detection reagents (Millipore, Billerica, MA). Quantification was performed by using the image-J software (National Institute of Health, NIH, Bethesda, MD).

### RNA Extraction and Quantitative Real-Time-Polymerase Chain Reaction (qRT-PCR)

Total RNA samples were extracted by using the TRIZOL (Invitrogen, Carlsbad, CA) according to the manufacturer’s instructions and previously reported (12). Briefly, we used DNase I (amplification grade, Invitrogen) to treat 1µg of total RNA before first-strand cDNA synthesis by using reverse transcriptase (Superscript II RT, Invitrogen). Then, PCR reactions were performed by using the ABI StepOne real-time PCR system (Applied Biosystems, Foster City, CA). For PCR, specific primers were used accordingly. The relative expression of IRAK2 was estimated by using the comparative Ct method. The following primers were used: IRAK2, forward, CCAGCCTGCAGGAGGTGTGTGG and reverse, CATCAAGGCTGGAATTGTCAAC; GAPDH, forward, AGCCACATCGCTCAGACAC and reverse, GCCCAATACGACCAAATCC.

### Flow Cytometry Analysis

Apoptosis was measured by using the FACScan flow cytometer (Becton Dickinson, Franklin Lakes, NJ) with combined-agent Apoptosis Detection Kit (BD Bioscience, Heidelberg, Germany). Annexin V, Fluorescein isothiocyanate (FITC), and 7-aminoactinomycin D (7AAD) were applied and analyzed following the manufacturer’s instruction. The cells were treated with or without 4Gy for 48 hours and collected by trypsinization. Cell pellets were resuspended in 400 µl 1X binding buffer and then stained with 5.0 µl annexin V-FITC as well as 2.5 µl 7-AAD for 20 min at room temperature in the dark and then analyzed *via* flow cytometry.

### Transient Overexpression

First, cells were plated in a 6-cm culture dish. The plasmid for human IRAK2 (Myc-DDK-tagged) ectopic expression was purchased from Origene (Rockville, MD). We transfected pCMV6-IRAK2 plasmid into OSCC cells by using Lipofectamine 2000^®^ (Invitrogen, Carlsbad, CA) and Opti-MEM medium, according to the manufacturer’s protocols. Cells were finally selected for stable clones by using the medium that contained 400μg/ml Geneticin (G418, Invitrogen).

### Transient Knockdown

The human IRAK2 shRNA sequence was purchased from the National RNAi Core Facility at Academia Sinica, Taiwan (clone ID: TRCN0000418431). Lentiviral constructs that expressed IRAK2-shRNA were subcloned into pLKO.1-puro plasmid, a lentiviral vector for cDNA expression (Sigma-Aldrich, St. Louis MO). All lentiviral vectors were transfected into 293T cells by using Lipofectamine 2000 reagent according to the manufacturer’s instructions. For lentiviral transduction, cells were treated with 8 μg/ml Polybrene (Sigma-Aldrich). Viral supernatants were added to the cell culture medium for 48 hours. The transduced cells were selected with 3 ug/ml puromycin (Gibco; Thermo Fisher Scientific, Inc., Waltham, MA, USA).

### Immunohistochemistry

Paraffin-embedded oral tumor sections were stained with an anti-IRAK2 antibody (monoclonal; Abnova, Taiwan) and then detected by using the Super Sensitive™ Polymer-HRP IHC detection system (Biogenex, San Ramon, CA), according to the manufacturer’s instructions. Antigens were retrieved by using EDTA buffer (pH9.0) at 100°C for 25 minutes. Then, the slides were incubated at room temperature with 1:50-diluted IRAK2 antibody for 1 hour, followed by washing with 1XTBS-T. Finally, the sections were incubated with diaminobenzidine (DAB) for 5 minutes to generate signals. The pathologist evaluated stained slides of individual patients. The staining intensity of IRAK2 over the cell membrane or cytoplasmic region was scored by using a scale ranging from 0–3 and percentages (0-100%). The score was a continuous variable, ranging from 0–300. The value was calculated by using the following formula: 1 × (percentage of weakly stained cells, i.e., 1+) + 2 × (percentage of moderately stained cells, i.e., 2+) + 3 × (percentage of strongly stained cells, i.e., 3+). The median IHC score of 110 was applied as a cut-off value to differentiate high or low expression.

### *In Vivo* Tumorigenesis

We used male 6-week-old athymic nude mice (BALB/cAnN.Cg-Foxn1nu/CrlNarl) for the *in vivo* xenograft experiment. Null vector OML1-R cells and stable IRAK2-transfected OML1-R cells (2 × 10^7^ cells) were suspended in 200 ul PBS. Then, these cells were injected subcutaneously into the left and right flanks of each mouse, respectively (n = 3 in each group). Tumor volume was monitored and quantified with a tumor volume growth ratio (final volume/initial volume). For exploring the role of IRAK2 in radiosensitivity *in vivo*, the control and IRAK2-overexpressed groups were designed (n = 3 in each group). IR treatment was started 40 days after cancer cells transplantation. As a similar IR protocol of cell irradiation (5), we delivered IR every four days. A total dose of 50 Gy was given in 10 fractions (Varian linear accelerator, US). All animal protocols were performed according to the instructions of the Institutional Animal Care and Use Committee of National Chung Cheng University (IACUC no.1060703).

### Statistical Analysis

All statistical analyses were performed by using the SigmaPlot software, version 10.0 (Systat Software Inc., San Jose, CA, USA) and SPSS (version 12.0; SPSS Inc., Chicago, IL, USA), accordingly. Continuous data were presented as mean ± standard deviation, and their statistically significant levels were calculated by using the Student’s t-test. Category data were analyzed by using the chi-square test. Time-to-event endpoints were estimated using the Kaplan-Meier plot, and the log-rank test was applied to assess curve differences between groups. Cox proportional regression analysis was used for univariate and multivariate analysis. All hazard ratios were provided with 95% confidence intervals to demarcate effective size. *P* values of less than 0.05 were defined as statistical significance.

## Results

### IRAK2 Affected the Sensitivity of OML1 and ML1-R Cells to IR

**Figure 1:**
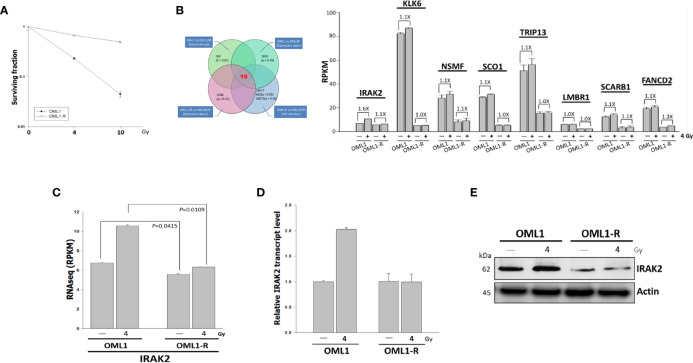
Higher IRAK2 expression was associated with a higher radiosensitivity in the context of parental (i.e., OML1) and radioresistant (i.e., OML1-R) OSCC cells. **(A)** After exposure to 0, 4, and 10 Gy IR, colony-formation assay confirmed that OML1-R cells were relatively radioresistant when compared with parental OML1 cells. **(B)** Venn diagram showed the number of genes with apparent expression change before and after irradiation in OML1 and OML1-R cells (left). Bar graphs displayed 19 genes were up-regulated in OML1 cells, using a filter criterion at least 1.5-fold change with P < 0.05. By setting a threshold of RPKM>2, we identified eight reliable transcripts that were largely differentially expressed between the OML1 and OML1-R cells. The graph showed relative fold change in gene expression: control *versus* IR-treated cells (right). **(C)** The RPKM value of IRAK2 expression was plotted for OML1 and OML1-R cells treated with 4 Gy. **(D)** qPCR and **(E)** Western blotting revealed that IRAK2 expression, including mRNA and protein levels, were pronouncedly elevated in parental OML1, but not OML1-R cells. Densitometry-derived values (bottom) were normalized with the control set as 1. β-actin served as the loading control for normalization.

Clonogenic assay confirmed a higher radiosensitivity of OML1 than that of OML1-R cells. When treated with the same dose of IR, especially 10 Gy, OML1-R cells exhibited a higher survival fraction than that of parental OML1 cells, suggesting that OML1-R is relatively resistant to IR treatment (**Figure 1A**). To identify genes whose expressions were altered after exposure to radiation, we utilized RNA-seq to assess the expression pattern of genes between paired parent (OML1) and radioresistant (OML1-R) cell lines treated with or without IR. By comparing the expression profiles of the two cell lines, we hypothesized that radiation exposure could activate genes responsible for the radiosensitivity process. By using reads per kilobase transcript per million mapped reads (RPKM) to estimate gene expression, we identified 19 genes that exhibited statistically significant differences of higher than 1.5-fold between OML1 and OML1-R cells after IR (**Figure 1B**). We further filtered out lowly expressed transcripts (i.e., an absolute cut-off value of RPKM < 2). As a result, eight genes were identified, including IRAK2, KLK6, NSMF, SCO1, TRIP13, LMBR1, SCARB1, and FANCD2 (**Figure 1B**). Of these, we found that IRAK2 showed the maximum fold change of gene expression (**Figure 1B**). Hence, we chose IRAK2 as our target for further functional analysis because it plays a vital role in regulating innate immunity (22) and may have great potential in predicting radiation response of OSCC cells. The data showed that the RPKM value of IRAK2 expression of the OML1-R was lower than that of OML1 cell lines whether control or IR treatment (**Figure 1C**). Real-time quantitative PCR (qPCR) and Western blotting analysis revealed that both mRNA and protein expressions of IRAK2 were up-regulated in irradiated OML1 cells compared with IR-treated OML1-R cells (**Figures 1D, E**).

### IRAK2 Overexpression Restored Radiosensitivity by Enhancing IR-Induced Cell Killing and Apoptosis in Radioresistant OML1-R Cells

**Figure 2:**
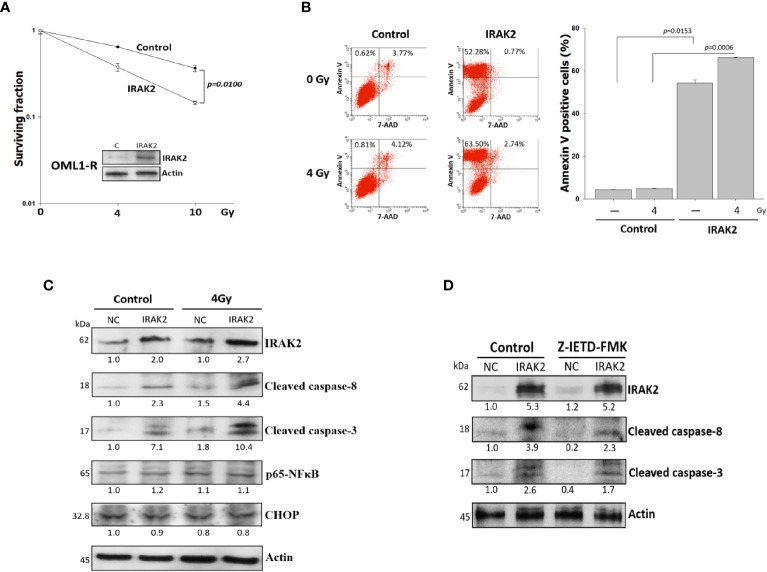
Overexpression of IRAK2 restored radiosensitivity *via* enhancing radiation-induced apoptosis in OML1-R cells. **(A)** Colony formation assay showed that IRAK2-overexpressed OML1-R cells restored their radiosensitivity when compared with that of control OML1-R cells (P = 0.0100). **(B)** Apoptosis-specific flow cytometry represented that overexpression of IRAK2 significantly enhanced apoptosis in OML1-R cells before (52.28% *vs.* 0.62%) and after (63.50% *vs.* 0.81%) 4-Gy IR. The histogram on the right represent Annexin V-positive staining enrichment. **(C)** In OML1-R cells, protein levels of apoptosis-related factors, i.e., cleaved caspase-8, cleaved caspase-3, CHOP, and p65-NF-κB, were elevated by the overexpression of IRAK2, especially after 4-Gy IR (Western blotting, 72 hours after IR). **(D)** Protein levels of IRAK2, cleaved caspase-8 and cleaved caspase-3 were analyzed for OML1-R cells treated with an IRAK2 overexpression followed by the pretreatment with caspase-8 inhibitors (50 mM Z-IETD-FMK) for 1 hour. Densitometry-derived values (bottom) were normalized with the control set as 1. β-actin served as the loading control for normalization.

To evaluate whether IRAK2 influences the sensitivity of radioresistant OSCC to IR, we overexpressed IRAK2 in OML1-R cells, which demonstrated an intrinsically low level of IRAK2. As shown in **Figure 2A**, IRAK2-overexpressed OML1-R cells exhibit a higher radiosensitivity than that of control OML1-R cells (P = 0.0100), suggesting a role of IRAK2 in the process of restoring radiosensitivity in radioresistant OSCC.

Apoptosis has been well known as a biological indicator for measuring cellular radiosensitivity (23). IRAK2-overexpressed OML1-R cells showed more apoptosis than that of control OML1-R cells, especially after 4Gy IR treatment; quantitative data for apoptosis rate were consistent with this phenomenon (**Figure 2B**).

IRAK2 is critical for apoptosis through FADD-dependent recruitment of caspase-8 activation (8) and the endoplasmic reticulum (ER) stress-induced IRE-1/CHOP signaling pathway (9). After 72 hours of exposure to 4-Gy IR, IRAK2-overexpressed OML1-R cells had higher levels of cleaved caspase-8 and caspase-3, but not NF-κB and CHOP, than that of control OML1-R cells (**Figure 2C**). To further confirm these results, we applied z-IETD-FMK, a caspase-8 inhibitor, to pretreat IRAK2-overexpressed OML1-R cells. Our results revealed that z-IETD-FMK attenuated the overexpression of IRAK2 -induced apoptosis, as shown by significant decreases in cleaved caspase-8 and cleaved caspase-3 (**Figure 2D**). These results simultaneously indicated that IRAK2 overexpression re-sensitizes OML1-R cells to IR treatment *via* enhancing caspase-8- and caspase-3-depended cell apoptosis.

### IRAK2 Knockdown Decreased OSCC Radiosensitivity and IR-Induced Apoptosis

**Figure 3:**
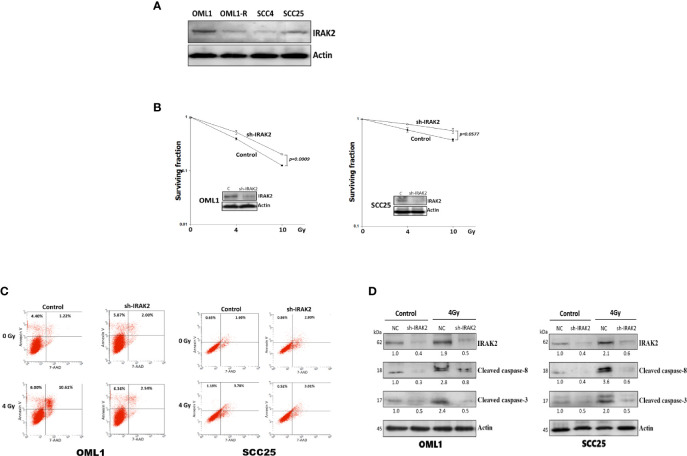
The knockdown of IRAK2 promoted resistance to IR-induced apoptosis in OML1 and SCC25 cells. **(A)** Endogenous IRAK2 expression in different OSCC cell lines, showing higher expressions of IRAK2 in OML1 and SCC25 than that of OML1-R and SCC4 cells. The values under bands represented the relative density that normalized to β-actin. **(B)** When compared with their control cells, post-irradiation colony formation rates were increased in IRAK2-knockdown OML1 (P = 0.0009) and SCC25 (P = 0.0577) cells. **(C)** Effect of radiation, IRAK2 shRNA or both on cell apoptosis in OML1 and SCC25 cell lines. Flow cytometry analysis using Annexin V and 7-AAD staining was performed to detect apoptotic cells. **(D)** IRAK2-shRNA transfection decreased the expressions of cleaved caspase-8 and caspase-3 in OML1 (left) and SCC25 (right) cancer cells. Densitometry-derived values (bottom) were normalized with the control set as 1. β-actin served as the loading control for normalization.

To further address whether IRAK2 is sufficient to induce apoptosis and alter cellular radiosensitivity, we quantified the expression of IRAK2 in four OSCC cell lines, finding that OML1 and SCC25 cells exhibited higher expression of IRAK2 (**Figure 3A**). IRAK2-specific shRNA was delivered into OML1 and SCC25 cells to knock down IRAK2 expression. Both IRAK2-knockdown OML-1 and SCC25 cells demonstrated higher survival rates after exposure to IR when compared with their control cells (P = 0.0009 and 0.0577, respectively; **Figure 3B**). To exam the apoptotic effects of IRAK2 shRNA combined with radiation in OML1 and SCC25 cell lines. The results revealed that in both cell lines, shRNA-IRAK2 cells were diminished radiation-induced apoptosis compared with their control cells after IR exposure (**Figure 3C**). IRAK2 silencing also decreased the expression of cleaved caspase-8 and -3 in both two types of OSCC cancer cells when compared with their control cells (**Figure 3D**). These data indicated that IRAK2 knockdown in OSCC cells strikingly attenuated radiosensitivity *via* inhibiting caspase-8/3-mediated apoptosis.

### IRAK2 Overexpression Enhanced IR-Induced Tumor Regression in Radioresistant OSCC Xenografts

**Figure 4:**
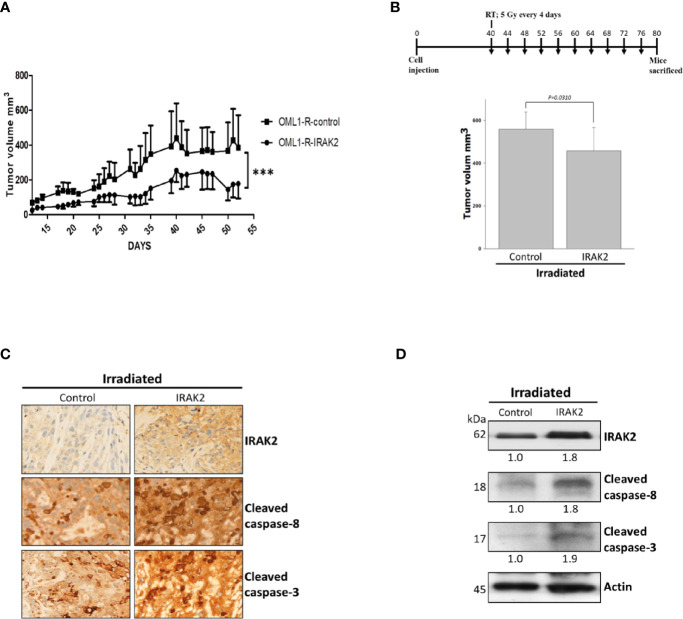
IRAK2 overexpression decreased OML1-R-generated *in vivo* tumor growth and enhanced radiosensitivity in the mice xenograft model. **(A)** In the mice xenograft model, IRAK2-overexpressed OML1-R-generated tumors had a relatively lower tumor growth rate than that of control OML1-R-generated tumors (P < 0.001). **(B)** Schema of cell injection and radiation treatments (upper panel). Since the 40th day after cancer cell injection, control and IRAK2-overexpressed mice were treated with RT per 4 days (i.e., a fraction size of 5 Gy by ten fractions to an accumulative dose of 50 Gy). The IRAK2-overexpressed mice had a smaller tumor volume than that of control mice at the time of radiotherapy (P = 0.0310; lower panel). **(C)** The expression of IRAK2, cleaved caspase-8, and cleaved caspase-3 in tumor tissues from control and IRAK2-overexpressed mice after radiotherapy were detected by immunohistochemical staining. **(D)** Immunoblotting analysis of the indicated proteins in lung tissues from control and IRAK2-overexpressed mice after irradiation. Densitometry-derived values (bottom) were normalized with the control set as 1. β-actin served as the loading control for normalization. Data were presented as mean ± SD. ‘***’ represented P < 0.001. All experiments were performed in triplicate.

To evaluate the radiosensitization potential of IRAK2 *in vivo*, we established a nude mice xenograft model that injected IRAK2-overexpressed OML1-R cells. IRAK2-overexpressed xenografts demonstrated an apparent reduction in tumor volume when compared with the control. IRAK2 overexpression alone inhibited tumor growth, indicating that IRAK2 may function as a tumor suppressor (**Figure 4A**). We then examined the effect of control and IRAK2-overexpressed mice that received a fraction size of 5 Gy every four days to a cumulative dose of 50 Gy, respectively. After exposure to RT, the tumor volume of IRAK2-overexpressed mice was statistically significantly decreased when compared with that of control ones, implicating that IRAK2 enhances the efficacy of IR treatment in radioresistant tumors (**Figure 4B**). The increased expressions of cleaved caspase-8 and cleaved caspase-3 were further confirmed by immunofluorescence staining and western blotting on tumor tissues in IRAK2-overexpressed xenografted mice treated with RT (**Figures 4C, D**).

### High IRAK2 Expression Was Associated With Favorable Local Control in Oral Cancer Patients

**Figure 5:**
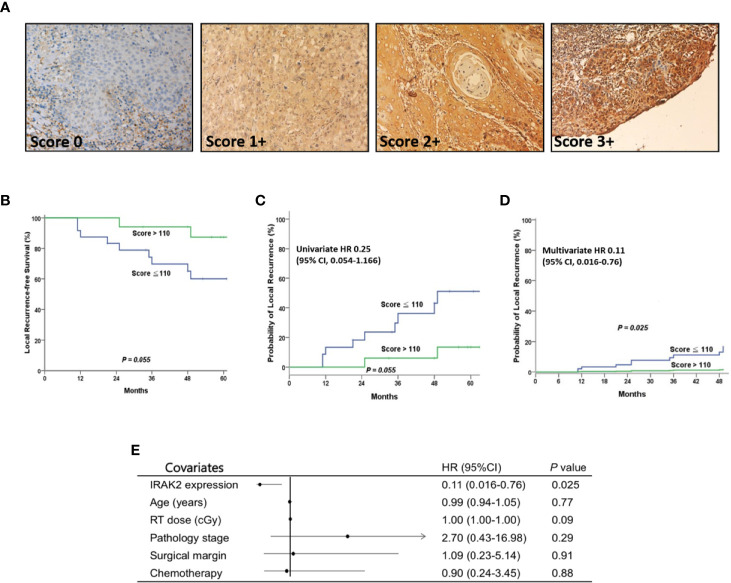
After postoperative radiotherapy, OSCC patients with higher IRAK2 expressions showed better local recurrence-free survival (LRFS) than those with lower IRAK2 expressions. **(A)** Representative micrographs demonstrated the immunohistochemical (IHC) scores of IRAK2 expression in oral squamous cell carcinoma. (magnification, x200). **(B)** Kaplan-Meier survival curves represented that patients with higher IRAK2 expressions (i.e., >110) seemly demonstrated better 5-year LRFS than that of patients with lower expressions (i.e., 87.4% *vs.* 60.0%; P = 0.055, a statistical trend). **(C)** Cox proportional hazard regression confirmed this observation (univariate HR 0.25; 95% CI, 0.054 - 1.166; P = 0.055, a statistical trend). **(D)** Remarkably, after multivariable analysis, the two groups showed a statistically significant difference in terms of post-irradiation LRFS (multivariate HR 0.11; 95% CI, 0.016 - 0.760; P = 0.025). **(E)** Forest plot of multivariate analysis, depending on the Panel **(D)** Note that seven factors were used for multivariate analysis of LRFS (Panel C): age, gender, pathological stage, RT dose, chemotherapy, the status of surgical margin, and the expression level of IRAK2.

Finally, we examined the expression of IRAK2 in 41 OSCC patient samples and summarized clinicopathologic factors in **Table 1**. No statistically significant correlation was found between the level of IRAK2 expression and other clinicopathological variables. Immunohistochemical staining showed IRAK2 expression in the cytoplasm and membrane of OSCC tumor samples (**Figure 5A**). Kaplan-Meier survival curves was performed to demonstrate that patients with higher IRAK2 expression (i.e., >110) were associated with better local recurrence-free survival than that of those patients with lower expressions (i.e., ≤110; P = 0.055; **Figure 5B**). Cox proportional hazard regression confirmed this observation (univariate HR, 0.25, being slight in favor of high expression; 95% CI, 0.054 - 1.166; P = 0.055; **Figure 5C**), particularly after multivariable analysis (multivariate HR, 0.11; 95% CI, 0.016 - 0.760; P = 0.025; **Figures 5D, E**). Note that seven factors were used for multivariable analysis of local recurrence: age, gender, pathological stage, radiotherapy dose, chemotherapy, the status of surgical margin, and expression level of IRAK2.

**Table 1:** Patient characteristics of 41 oral cancer patients according to the expression level of IRAK2.

		IRAK2				
		Low expression (n = 24)		High expression (n = 17)		*P* value
Age (years)	(mean ± SD)	51.3 ± 11.4		52.7 ± 10.1		0.70
RT dose (cGy)	(mean ± SD)	6338.3 ± 1378.4		6559.4 ± 1284.8		0.61
Gender	Male	23	(96%)	16	(94%)	0.80
	Female	1	(4%)	1	(6%)	
Clinical stage	I	8	(33%)	4	(24%)	0.88
	II	11	(46%)	9	(53%)	
	III	2	(8%)	1	(6%)	
	IVA/B	3	(13%)	3	(18%)	
Pathologic stage	I	15	(63%)	7	(41%)	0.18
	II	9	(38%)	10	(59%)	
Surgical margin	<1 mm	6	(25%)	1	(5.9%)	0.21
	≧1~ ≦5 mm	18	(75%)	16	(94.1%)	
Lymphovascular space invasion	No	23	(95.8%)	14	(82.4%)	0.29
	Yes	1	(4.2%)	3	(17.6%)	
Perineural invasion	No	22	(91.7%)	13	(76.5%)	0.18
	Yes	2	(8.3%)	4	(23.5%)	
Chemotherapy	No	17	(71%)	10	(59%)	0.42
	Yes	7	(29%)	7	(41%)	

SD, standard deviation; RT, radiotherapy.

According to the principle of surgical oncology and our treatment policy, re-resection was the first treatment of choice for patients who had close-margin (i.e., ≦5 mm) pathology stage I-II OSCC. However, for those patients who had anatomic difficulty on re-resection and who had refusal of re-operation, salvage therapy of radiotherapy with or without chemotherapy was the alternative treatment choice, as that indicated for the above 41 patients.

## Discussion

Radioresistance remains a major obstacle for the radiotherapy treatment of OSCC, leading to post-irradiation recurrence and poor clinical outcomes (24, 25). However, so far, few biomarkers are available for identifying potential responders to RT. Based on RNA-seq analysis, we determined that IRAK2 might be an IR-responsive gene whose expression differed significantly between radiosensitive and radioresistant OSCC cells. We found that IRAK2 was downregulated in a radioresistant OML1-R cell line when compared with its parental OML1 cell line. We further examined the expression of IRAK2 in IR-sensitive OML1 and IR-resistant OML1-R cells and showed that the mRNA and protein expression levels of IRAK2 were increased mainly in OML1 cells after IR exposure; however, there was no difference in IRAK2 level between unirradiated and irradiated OML1-R cells, suggesting that the loss of IRAK2 might be an indicator or possibly contribute to mechanisms of radiation resistance. To further clarify the role of IRAK2 in RT, we overexpressed IRAK2 in OML1-R cell lines. Enhanced IRAK2 expression in OML1-R led to decreased colony formation after IR. Conversely, knocking down IRAK2 increased the post-irradiation survival of OSCC cells. Besides, *in vivo*, nude mice exposed to RT had smaller tumors after they were injected with IRAK2-overexpressed OML1-R cells. These sets of data strongly suggested that IRAK2 may serve as a potential therapeutic molecular marker for enhancing radiosensitivity and reversing radioresistance. Therefore, we confirmed that IRAK2 acts as a potential regulator of radiation sensitivity for OSCC.

IRAK2, an immune-responsive protein kinase, is a transducer for the IL1/TLR signaling cascade (6, 26, 27). Recent evidence suggests that TLR-dependent mechanisms contribute to radiation-induced anticancer immunity through the induction of genes associated with programmed cell death (28, 29). Mechanistically, TLR is connected through the interaction of an adaptor molecule (i.e., MyD88), which recruits FADD and caspase-8, leading to the activation of caspase-3 to trigger the apoptotic process subsequently (30). For example, BEAS-2B cells (which were derived from human bronchial epithelium transformed) were treated with a TLR3 agonist [i.e., poly(I:C)]; this manipulation was found to induce apoptosis through the interaction of MyD88 with FADD and caspase-8 (31). Besides, TLR2 was also found to potentiate the MyD88-induced caspase-8 apoptotic pathway in human kidney epithelial 293 cells (32). As a result, IRAK2 demonstrated a mediator of MyD88-dependent signal transduction activation *via* TLRs (6, 33).

IRAK2 also has been reported to be associated with signaling cell death (34). It could induce apoptosis in bacteria-infected macrophages by targeting the FADD/caspase-8 death signaling pathway (35, 36). Moreover, IRAK2 is required for ER stress-induced increases in IRE1 and CHOP expression, which transduces the death signal in ER stress-mediated apoptosis (9). In the present study, we found that overexpression of IRAK2 significantly increased the apoptotic rate in response to IR through cleavage activation of caspase 8 and caspase 3 in irradiated cells, whereas in the presence of z-IETD-FMK (caspase-8 inhibitor) could significantly decrease IRAK2-induced caspase-8 and caspase-3 cleavage. It suggested that IRAK2-induced apoptosis is dependent on the caspase-8 activation.

On the other hand, IRAK2 knockdown diminished the caspase-8 and caspase-3-mediated apoptosis. These findings indicated that IRAK2 might be a superior target for radiosensitization, triggering apoptosis mainly through the activation of caspase-8 and caspase-3 to increase and restore the sensitivity of radioresistant cells to IR-induced cell killing. However, the significant functional role of IRAK2 in mediating apoptosis and anticancer immunity by TLR signaling needs to be further investigated.

Since few reports have discussed the role of IRAK2 in solid tumors (37), we further evaluated the clinical significance of IRAK2 expression in 41 pathological stage I-II OSCC patients who underwent postoperative radiotherapy. Patients with high IRAK2 expression have been demonstrated to be better local control rates than those with low IRAK2 expression. The limitations of the present study are as follows: the nature of retrospective study design and a relatively small case number. Thus, further studies with a large sample size are required to confirm our results. Taken together, we found that IRAK2 was downregulated in radioresistant OSCC cells. We further determined that enhancing IRAK2 activity led to increasing/restoring radiosensitivity in radioresistant OSCC *in vitro* and *in vivo* – and vice versa – operating through a caspase-8/3-dependent apoptosis mechanism. Our data suggested that IRAK2 may affect both the developments of intrinsic and irradiation-acquired radioresistance.

The present study is the first report stating that IRAK2 activation may be associated with modulation of radiosensitivity. IRAK2 may facilitate the process of radiation immunity by inducing cancer cells to undergo apoptosis.

## Conclusions

In the present study, we have established a proof-of-concept platform *in vivo* and *in vitro* that the potential of IRAK2 may serve as both predictive and therapeutic biomarkers for estimating and manipulating radiation sensitivity in OSCC. Overexpressing IRAK2 increases and restores radiosensitivity in intrinsic and treatment-acquired radioresistant OSCC, respectively. Clinically, high IRAK2 expression predicts better local control in irradiated OSCC patients. However, the real-world clinical treatment utilization of IRAK2 in patients with OSCC remains unknown. Accordingly, further research in targeted gene therapy techniques to assess their efficacy and safety in OSCC is required.

## Data Availability Statement

The datasets presented in this study can be found in online repositories. The names of the repository/repositories and accession number(s) can be found below: GEO Series, accession number GSE165585.

## Ethics Statement

The studies involving human participants were reviewed and approved by Dalin Tzu Chi Hospital, Buddhist Tzu Chi Medical Foundation, approved number: B10604021. The patients/participants provided their written informed consent to participate in this study. The animal study was reviewed and approved by Animal protocols obeyed the instructions of the Institutional Animal Care and Use Committee of National Chung Cheng University (IACUC no.1060703).

## Author Contributions

C-CY, R-IL, H-YL, and F-CH performed the experiments. L-CC, L-WH, C-HC, and H-JY collected patient samples. W-YC, C-AC, C-LC, M-SL, and MC performed data analysis. MC, H-YL, and S-KH designed the experiments. C-CY, H-YL and S-KH wrote the manuscript. All authors contributed to the article and approved the submitted version.

## Funding

This study was supported by grants from the Dalin Tzu Chi Hospital, Buddhist Tzu Chi Medical Foundation (DTCRD105(2)-E-09, DTCRD106 (2)-E-17, DTCRD107(2)-I-13). This study was also supported by the Ministry of Science and Technology, Taiwan (MOST 108-2314-B-303-001), and Taipei City Hospital Zhongxing Branch (TPCH-107-031).

## Conflict of Interest

The authors declare that the research was conducted in the absence of any commercial or financial relationships that could be construed as a potential conflict of interest.
